# Supplementary material for: Long-Term Fate and Efficacy of a Biomimetic (Sr)-Apatite-Coated Carbon Patch Used for Bone Reconstruction
Source: J Funct Biomater. 2023 Apr 26;14(5):246. doi: 10.3390/jfb14050246 (PMC10218964; doi:10.3390/jfb14050246)
Supplement: Supplementary file 1 [file jfb-14-00246-s001.zip › jfb-2320101-supplementary.pdf]

Supplementary Information

Long term fate and efficacy of biomimetic apatite-coated carbon patch used for bone reconstruction

Florian Olivier, Christophe Drouet, Olivier Marsan, Vincent Sarou-Kanian, Samah Rekima, Nadine Gautier, Franck Fayon, Sylvie Bonnamy and Nathalie Rochet

Figure S1. Illustration of the segmentation method used to detect carbon material from histological slides. Microscopic images before (left side) and after segmentation (right side) of carbon (red) versus tissue (green).

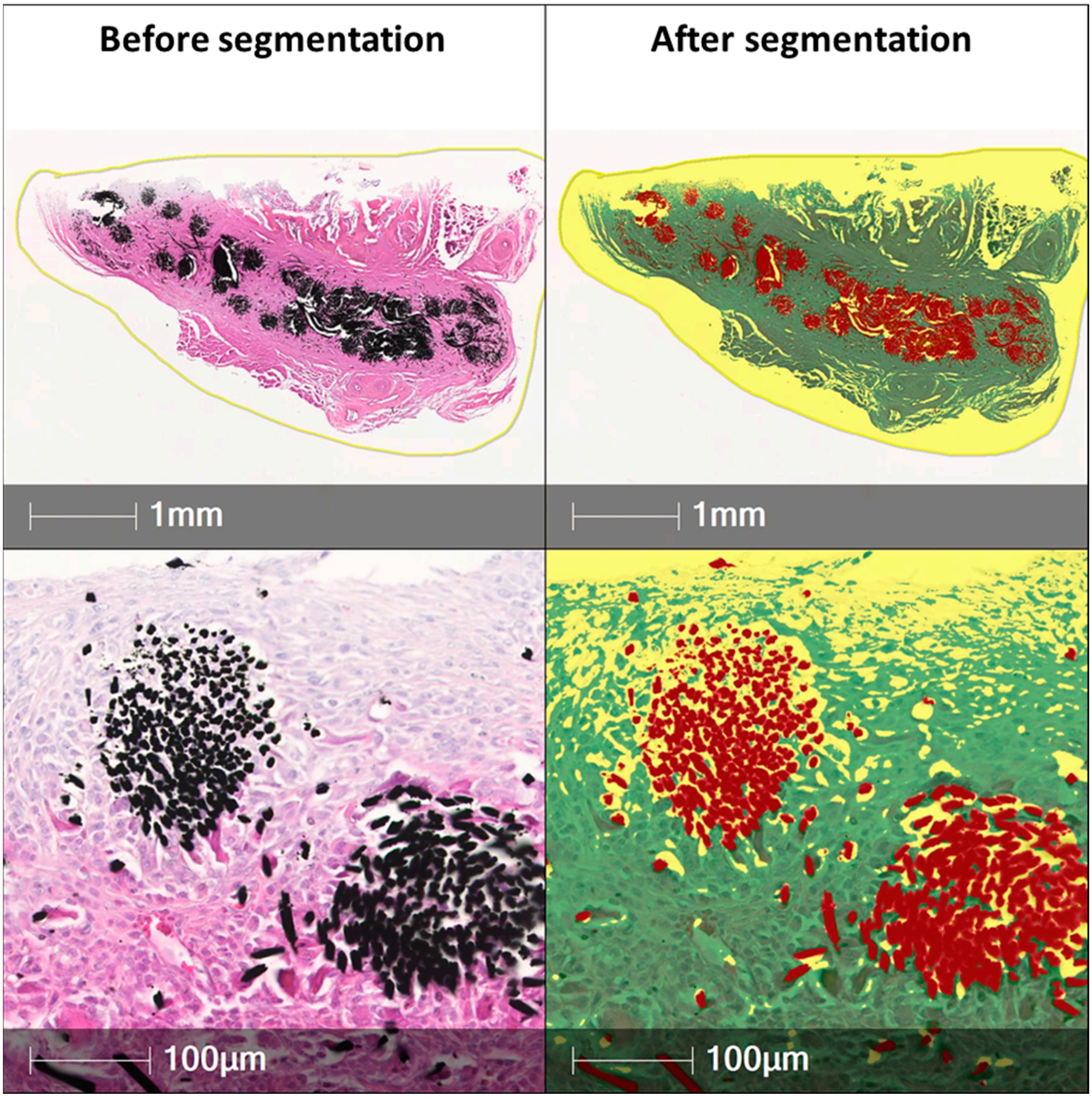

Figure S2. Raman spectra of the newly-formed bone from several punctual analyses for the three groups tested *in vivo* (control, ACC/CDA and ACC/10Sr-CDA). The similarity in position and width of the $\nu_1(PO_4)$ band is in particular highlighted.

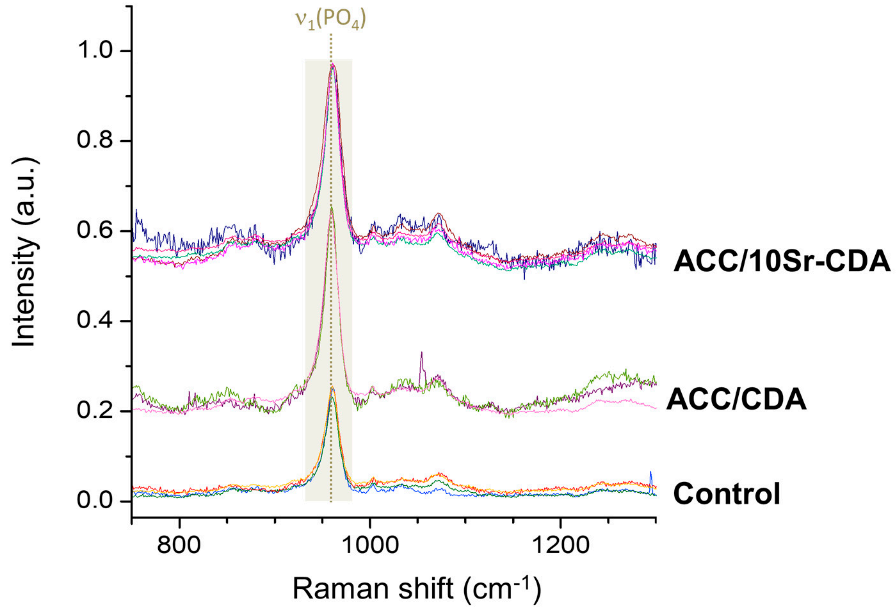

Table S1. Raman band characteristics after curve-fitting for different depths Z of analysis.

Sample positioning along Z axis	Remarkable peak assignments	Peak position	Peak height	Gaussian/Lorentzian mixing parameter (0 = L; 1 = G)	Peak width	Peak area
Z = 0 µm		824.2	192.9	1	18.3	3748.0
	proline	850.2	663.7	0.25	19.9	18007.8
		859.9	222.1	0.97	9.4	2262.4
	hydroxyproline	875.1	494.6	1	22.4	11804.6
		893.7	273.6	0.32	15.2	5718.9
		923.9	742.2	0.25	39.5	40231.4
	v1(PO4) am	955.3	2043.3	0.61	28.0	71030.3
	v1(PO4) ap	961.7	6230.4	0.44	13.2	109187.0
		1003.6	665.7	0	8.3	8560.7
		1035.0	1067.2	0.14	46.4	71267.7
	v1(CO3)	1071.6	538.4	0.51	21.1	14721.0
		1085.1	478.7	1	50.0	25476.2
		1184.4	137.9	1	26.9	3948.7
		1206.7	257.9	0.4	13.2	4603.5
	amide III	1241.4	359.3	1	23.8	9108.9
	amide III	1270.1	798.0	0.11	48.3	56048.8
	amide III	1305.1	432.6	1	20.3	9327.4
		1385.9	315.3	1	14.7	4933.4
		1402.7	212.3	0.5	22.1	6082.4
	CH2	1441.6	719.6	0.36	38.2	36509.5
	CH2	1453.4	799.7	1	32.9	28030.5
Z = 200 µm	proline	854.7	479.5	0.45	27.0	16876.0
	hydroxyproline	878.8	179.4	1	15.2	2896.5
	v1(PO4) am	946.1	1103.3	0.03	17.9	30214.2
	v1(PO4) ap	960.5	6160.1	0.87	15.9	110582.4
		1039.7	430.2	1	45.7	20943.4
	v1(CO3)	1072.1	792.0	0.79	20.9	19223.4
		1096.7	337.6	1	37.2	13381.1
	amide III	1241.8	334.7	0.57	22.4	9544.1
	amide III	1271.2	376.5	0	30.8	17709.7
		1340.8	3591.2	0.07	0.0	153.8
		1389.5	202.9	0.42	29.2	7873.3
		1422.9	342.3	0	24.8	12880.2
	CH2	1448.4	581.8	0.77	22.7	15354.4
	CH2	1464.4	318.8	1	21.4	7263.3
Z = 400 µm		814.8	329.8	0.43	17.9	7707.64
		833.6	282.4	1	14.0	4221.76
	proline	853.0	735.7	1	18.2	14235.39
	hydroxyproline	875.2	499.8	1	21.8	11609.33
	v1(PO4) am	953.9	3724.9	0.41	21.7	108387.29
	v1(PO4) ap	963.3	4157.4	1	14.6	64414.71
		1004.5	241.3	0.48	7.4	2364.74
		1032.4	630.6	0.08	17.0	16206.71
		1049.2	401.5	1	17.4	7429.4
	v1(CO3)	1072.0	1393.9	0.45	22.4	41465.02
		1097.1	465.8	0.55	37.1	21983.41
		1125.5	70.3	1	9.4	703.27
	amide III	1243.6	527.3	0.47	23.9	16577.44
	amide III	1272.6	391.0	0.6	24.3	11924.97
		1385.1	256.3	0.82	19.0	5570.12
		1401.6	201.2	1	11.0	2347.33
		1423.4	575.5	0	37.8	32388.66
	CH2	1451.7	621.1	1	26.4	17465.01
	CH2	1463.2	430.0	1	27.5	12586.23

Table S1. (continued)

Sample positioning along Z axis	Remarkable peak assignments	Peak position	Peak height	Gaussian/Lorentzian mixing parameter (0 = L; 1 = G)	Peak width	Peak area
**Z = 600 µm**		814.4	100.3	1	9.2	981.04
		827.1	61.2	0.81	13.0	916.45
	proline	854.3	538.7	0	30.9	24756.13
	hydroxyproline	878.7	258.1	1	14.0	3842.8
	v1(PO4) am	947.3	1210.0	0.81	20.8	29041.57
	v1(PO4) ap	961.0	6036.8	0.91	16.1	107412.78
		1037.1	435.0	0.03	33.7	22123.26
	v1(CO3)	1072.2	1072.8	0	27.1	44575.56
		1102.6	182.1	1	35.7	6912
	amide III	1242.9	355.3	0.82	24.9	10171.55
	amide III	1272.3	264.5	0.53	25.7	8725.19
		1375.2	411.5	1	19.2	8415.8
		1402.5	841.4	0.72	23.0	23117.12
		1422.1	273.4	0.32	17.0	6429.51
	CH2	1450.7	625.1	0.79	30.2	21827.96
	CH2	1467.3	242.5	0.35	26.4	8577.78
**Z = 800 µm**		831.9	347.6	0.49	50.0	21525.27
	proline	855.8	515.9	0.88	19.8	11413.19
	hydroxyproline	879.3	560.3	0.97	17.6	10628.11
	v1(PO4) am	946.4	1163.2	0.61	18.7	27214.18
	v1(PO4) ap	960.5	6092.0	0.81	15.9	111854.21
		1003.7	260.1	1	8.5	2346.54
		1040.9	516.6	1	37.5	20608.02
		1058.1	139.2	1	15.6	2317.35
	v1(CO3)	1071.0	671.4	1	15.6	11178.23
		1083.5	586.6	0	28.8	25871.29
		1104.5	191.3	0	31.3	9175.64
	amide III	1242.9	315.4	0.71	21.6	8195.04
	amide III	1271.6	323.9	0	25.4	12638.65
		1342.9	137.5	1	8.2	1195.84
		1387.5	267.2	1	25.1	7138.97
		1404.0	183.8	1	9.7	1898.43
		1421.5	413.9	0	25.7	16107.66
	CH2	1450.2	574.3	0.37	31.0	23736.17
	CH2	1458.2	382.4	1	31.1	12675.06
**Native cortical bone**		814.0	170.4	0	16.2	4135.83
	proline	853.3	404.5	0	22.1	13508.32
	hydroxyproline	877.1	178.0	1	18.3	3468.73
	v1(PO4) am	951.1	1772.1	0.77	25.9	53638.68
	v1(PO4) ap	960.5	5214.0	0.88	15.5	90744.63
		1002.9	254.1	1	6.3	1695.85
		1033.1	286.6	0.35	18.1	7152.23
	v1(CO3)	1072.4	910.5	0	28.8	40252.93
		1103.4	138.8	0	14.2	3050.87
		1202.4	91.3	1	6.3	616.14
	amide III	1242.2	334.6	1	21.5	7667.61
	amide III	1270.5	329.1	0.02	31.8	15898.97
		1420.9	302.8	0	50.0	22138.19
	CH2	1455.0	534.6	1	29.6	16825.86
	CH2	1470.7	8521.2	0.03	0.1	74.85
